# Supplementary material for: Selective Role of TNFα and IL10 in Regulation of Barrier Properties of the Colon in DMH-Induced Tumor and Healthy Rats
Source: Int J Mol Sci. 2022 Dec 9;23(24):15610. doi: 10.3390/ijms232415610 (PMC9779473; doi:10.3390/ijms232415610)

Original images supporting the blot results reported in the article

Western blotting of the colon tissues

From left to right:

the 1st band – control (proximal), the 2nd - TNFα (proximal), the 3rd - IL10 (proximal), the 4th - excluded data*;

the 5th band - control (distal), the 6th - TNFα (distal), the 7th - IL10 (distal), the 8th - excluded data*.

The samples, that are presented in the Figures 3 and 4, marked with ↑ below the photos.

*The excluded data belong to IL6 (4th and 8th bands), they were not included in the material of this article.

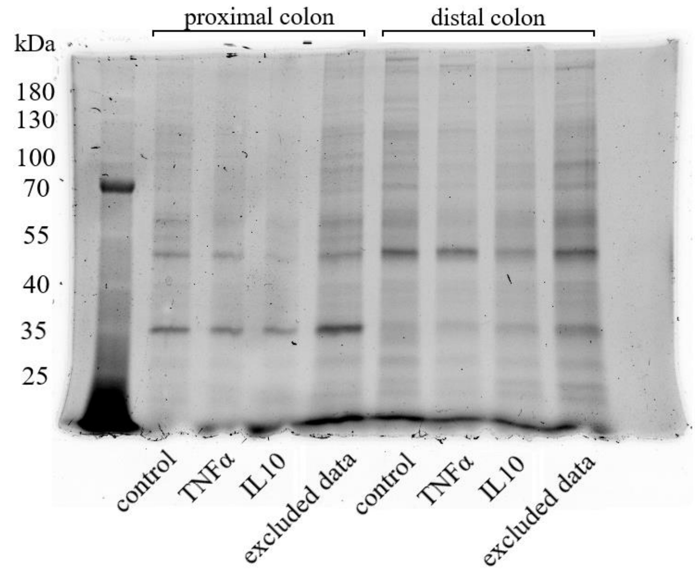

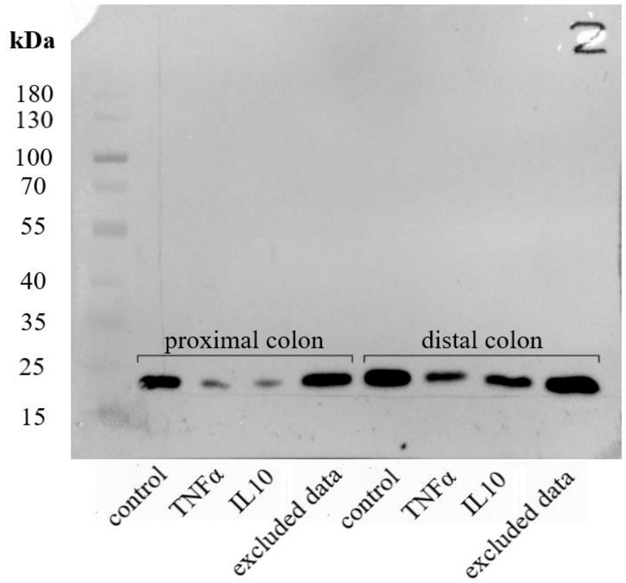

Claudin-1

Stain-free gel **Membrane**

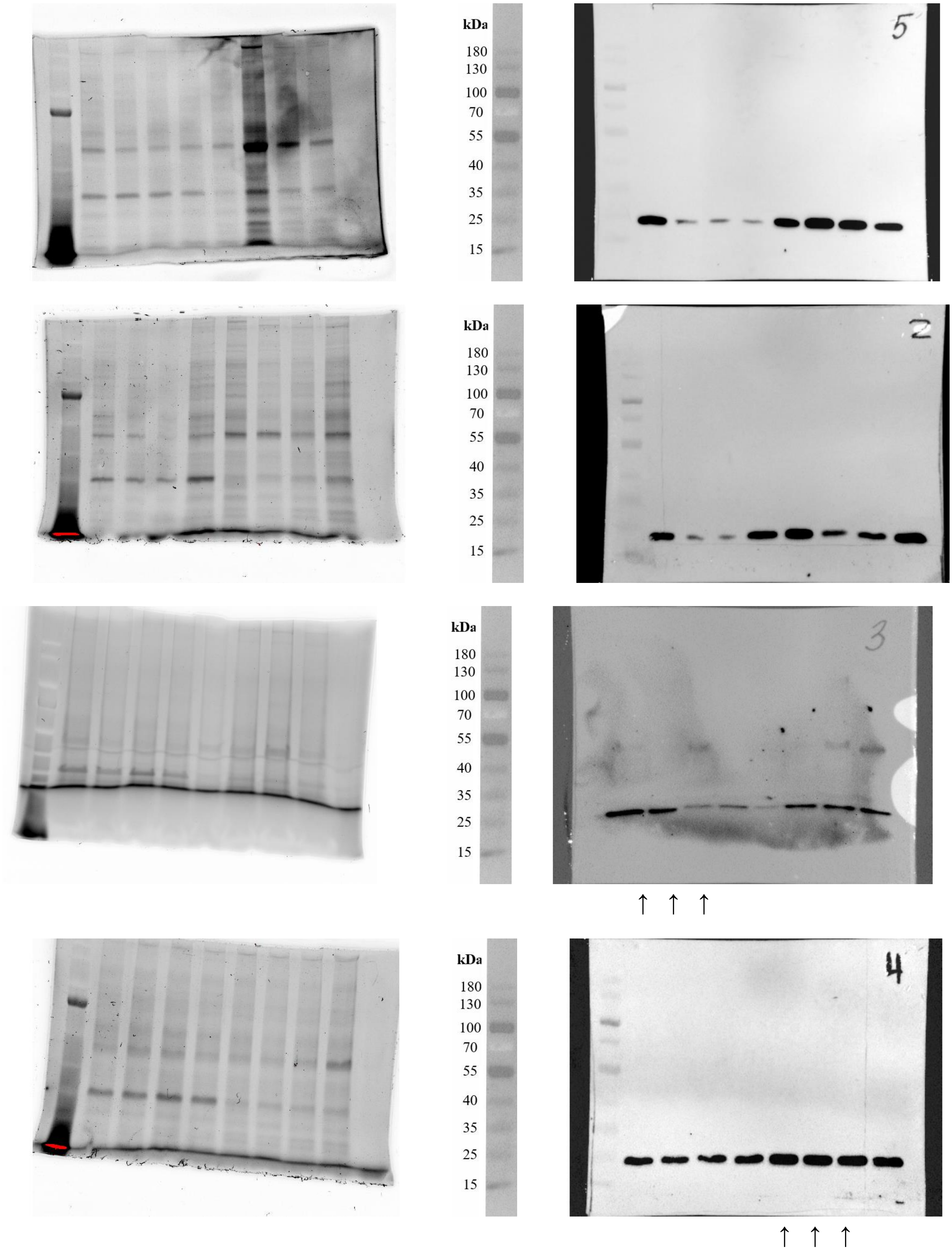

Claudin-2

Stain-free gel **Membrane**

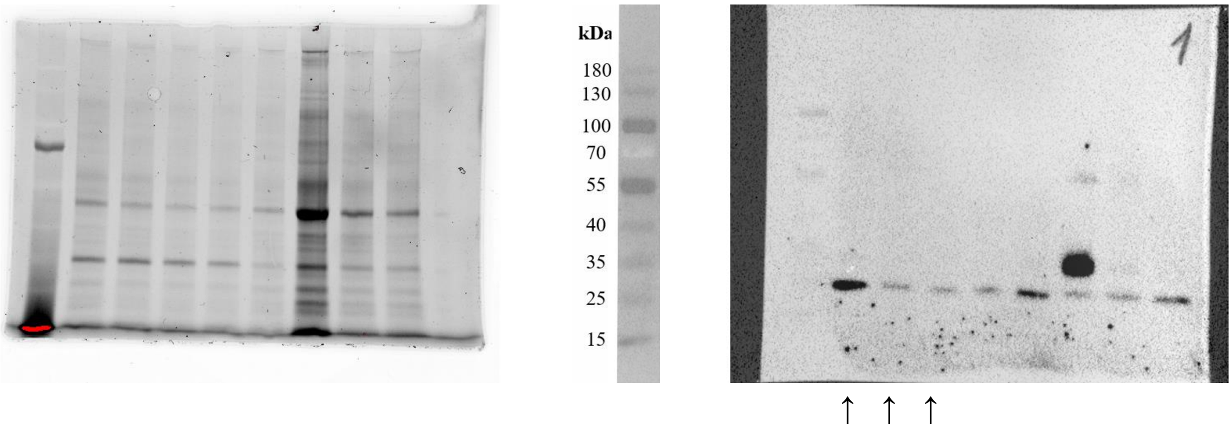

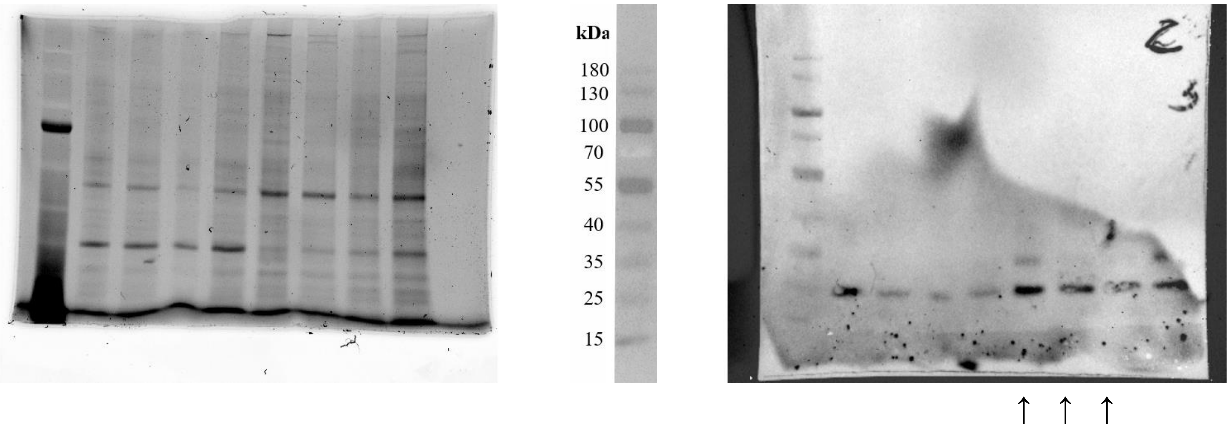

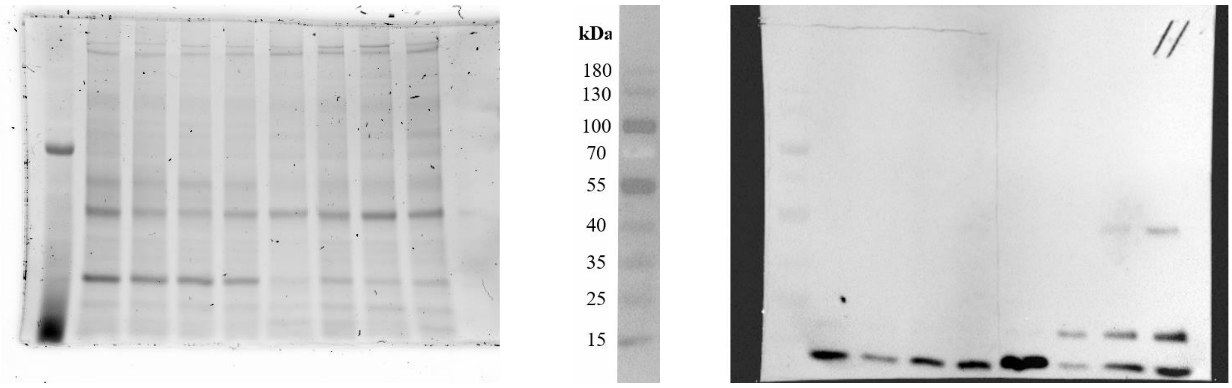

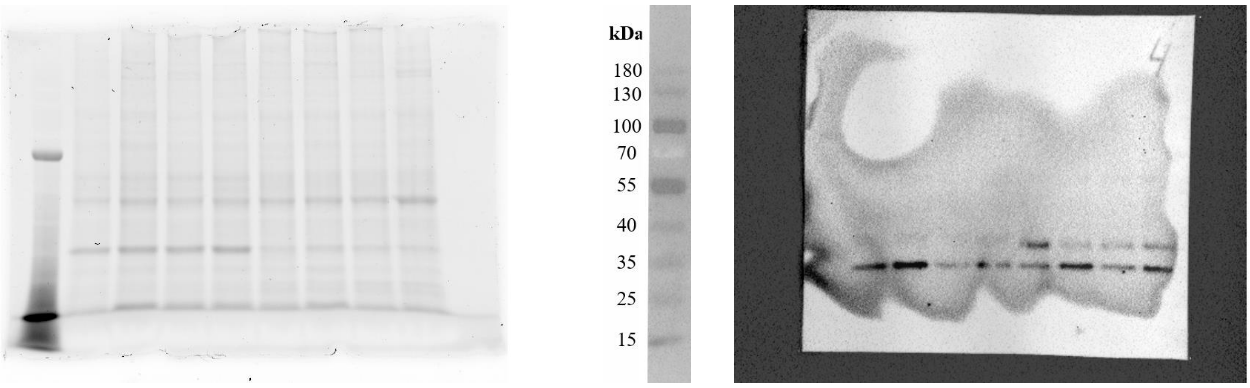

Claudin-3

Stain-free gel | Membrane

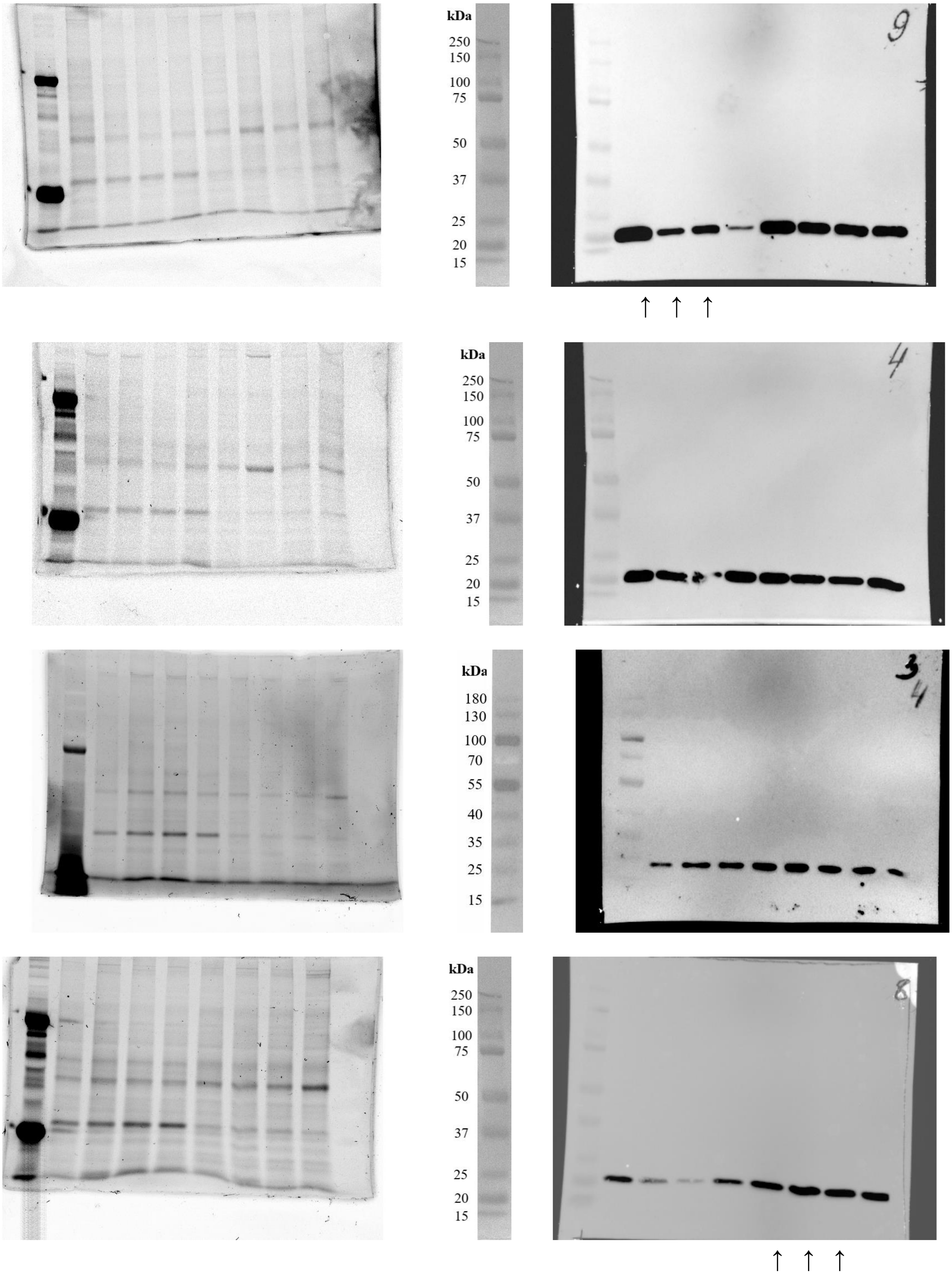

Claudin-4

Stain-free gel **Membrane**

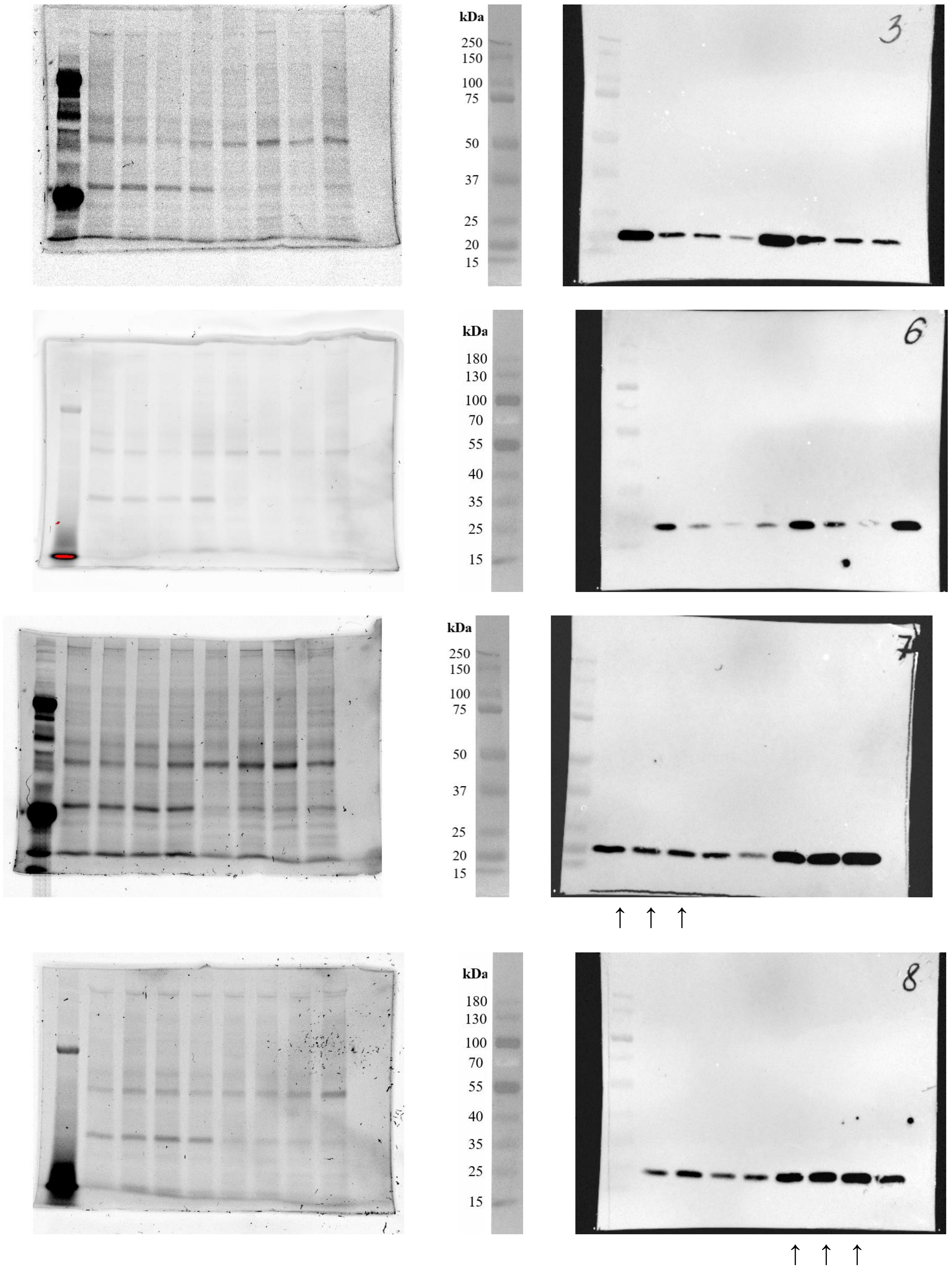

Occludin

Stain-free gel | Membrane

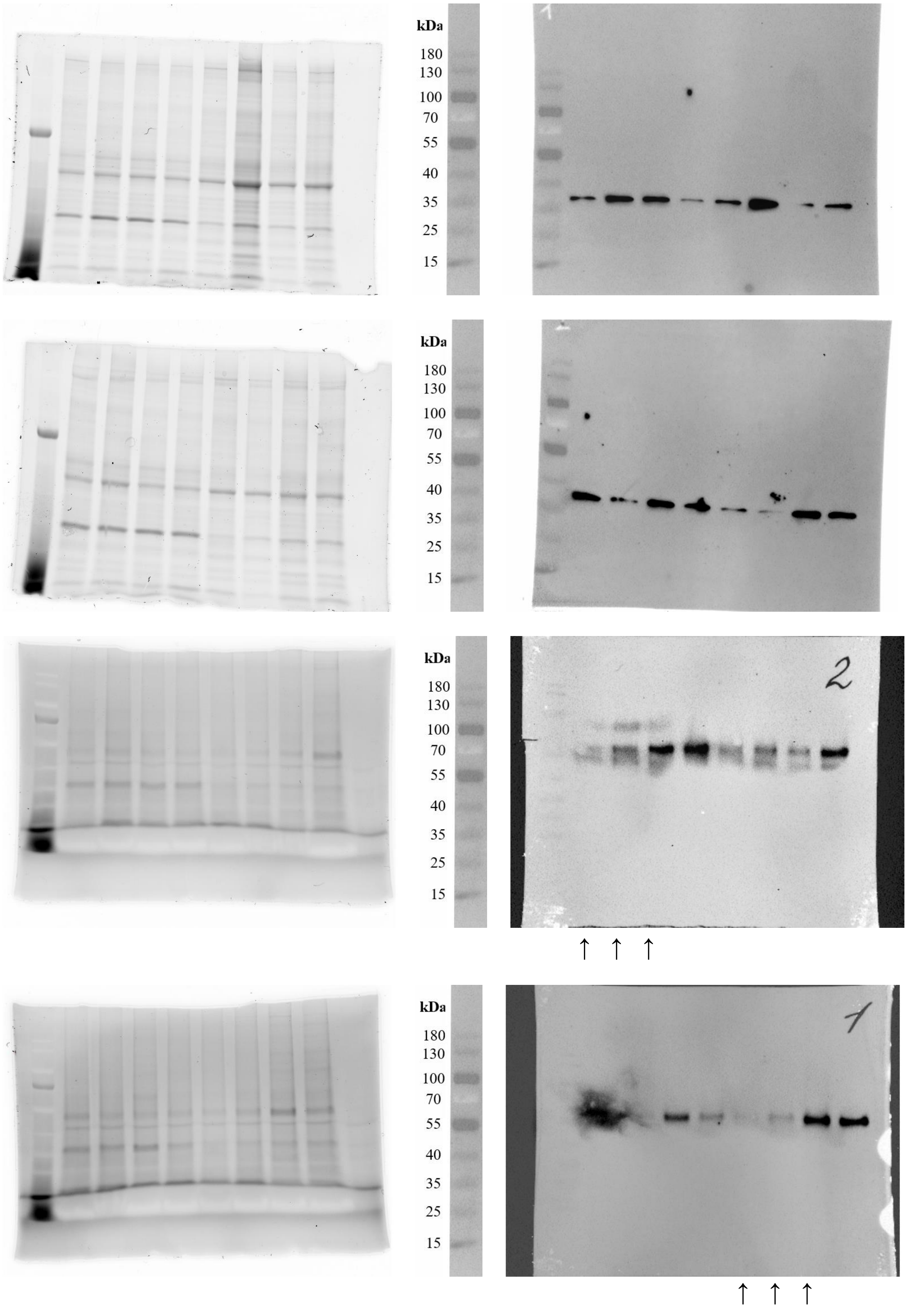

Tricellulin

Stain-free gel **Membrane**

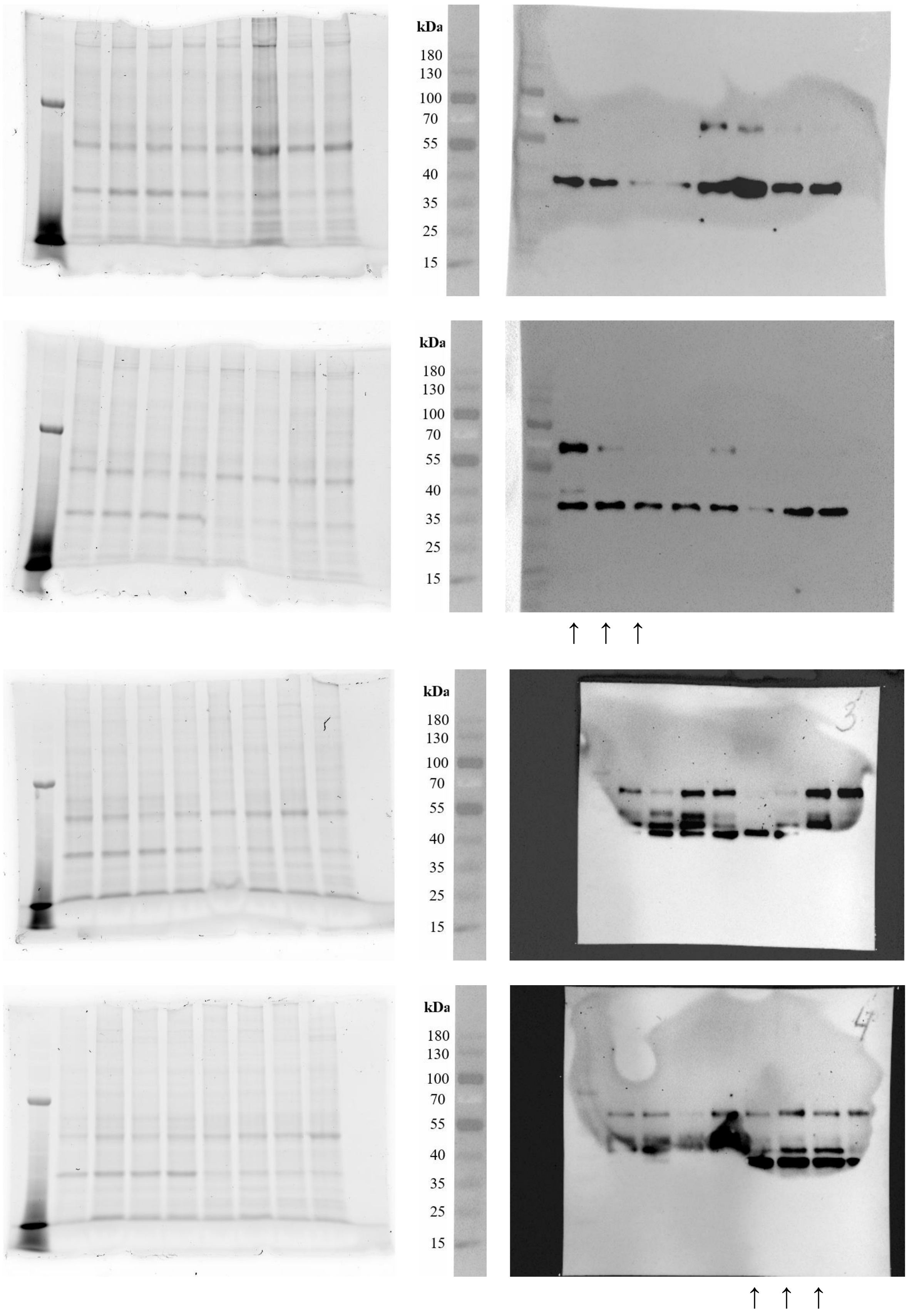

Supplement: Supplementary file 1 [file ijms-23-15610-s001.zip › ijms-2031339-supplementary.pdf]
